# Differences between diabetic and non-diabetic patients with community-acquired pneumonia in primary care in Spain

**DOI:** 10.1186/s12879-019-4534-x

**Published:** 2019-11-15

**Authors:** Loreto Arias Fernández, Jacobo Pardo Seco, Miriam Cebey-López, Ruth Gil Prieto, Irene Rivero-Calle, Federico Martinon-Torres, Ángel Gil de Miguel, on behalf of NEUMOEXPERTOS group, F. Martinón-Torres, D. Vargas, E. Mascarós, E. Redondo, J. L. Díaz-Maroto, M. Linares-Rufo, A. Gil, J. Molina, D. Ocaña, I. Rivero-Calle

**Affiliations:** 1 Epidemiology of Infectious Diseases, Rey Juan Carlos University, Avda. Atenas s/n, CP, 28922 Alcorcón, Madrid Spain; 2 Genetics, Vaccines, Infections and Pediatrics Research Group (GENVIP), Healthcare Research Institute of Santiago de Compostela, Santiago de Compostela, Spain; 3 Spa Genetics, Vaccines, Infections and Pediatrics Research Group (GENVIP), Healthcare Research Institute of Santiago de Compostela, Santiago de Compostela, Spain; 4 Area of Preventive Medicine & Public Health, Rey Juan Carlos University, Madrid, Spain

**Keywords:** Diabetes, Community-acquired pneumonia, Primary care, Risk factors

## Abstract

**Background:**

Diabetes is one of the underlying risk factors for developing community-acquired pneumonia (CAP). The high prevalence of diabetes among population and the rising incidence of this illness, converts it as an important disease to better control and manage, to prevent its secondary consequences as CAP.

The objective of this research is to describe the characteristics of the patients with diabetes and the differences with the no diabetes who have had an episode of CAP in the context of the primary care field.

**Methods:**

A retrospective, observational study in adult patients (> 18 years-old) who suffer from CAP and attended at primary care in Spain between 2009 and 2013 was developed using the Computerized Database for Pharmacoepidemiological Studies in Primary Care (BIFAP). We carried out a descriptive analysis of the first episodes of CAP, in patients with or without diabetes as comorbidity. Other morbidity (CVA, Anaemia, Arthritis, Asthma, Heart disease, Dementia, Depression, Dysphagia, Multiple sclerosis, Epilepsy, COPD, Liver disease, Arthrosis, Parkinson’s disease, Kidney disease, HIV) and life-style factors were also included in the study.

**Results:**

A total of 51,185 patients were included in the study as they suffer from the first episode of CAP. Of these, 8012 had diabetes as comorbidity. There were differences between sex and age in patients with diabetes. Patients without diabetes were younger, and had less comorbidities including those related to lifestyles such as smoking, alcoholism, social and dental problems than patients with diabetes.

**Conclusions:**

Patients who developed an episode of CAP with diabetes have more risk factors which could be reduced with an appropriate intervention, including vaccination to prevent successive CAP episodes and hospitalization.

The burden of associated factors in these patients can produce an accumulation of risk. Health care professional should know this for treating and control these patients in order to avoid complications. Diabetes and those other risk factors associated could be reduced with an appropriate intervention, including vaccination to prevent the first and successive CAP episodes and the subsequent hospitalization in severe cases.

## Background

According to the World Health Organization (WHO), lower respiratory tract infections are the third leading cause of death in the world [1]. Specifically, CAP is ranked as the fifth cause of mortality global [2] and in 2013, pneumococcal pneumonia accounted for more than 20% of these cases [3]. Furthermore, in addition to pneumococcal pneumonia being an important cause of mortality, lower respiratory tract infections represent the third leading cause of lost years of life after adjusting for disability [4].

The probability of hospitalization in patients suffering from community-acquired pneumonia (CAP) with an underlying comorbidity such as a cardiac, respiratory or metabolic pathology is 73 times higher than in patients without a comorbidity [5]. The detection of these comorbidities is important for prevention, such as pneumococcal vaccination, and to prevent excessive hospitalization. Diabetes is one of these comorbidities [6]. In Europe, the prevalence of diabetes is 8.8%, and this number is projected to increase [7]. Specifically, the prevalence of type 2 diabetes in people over 18 years of age in Spain is 13.8% [8].

Although diabetes is detected and controlled primarily through primary care (PC) clinics [9], studies on the burden of CAP in diabetic patients in the field of PC are very scarce [10], as the focus is usually on the hospital setting [11].

Given the difficulty of immunizing adult patients [12], the PC consultation is a favourable environment for detecting patients who may be good candidates for vaccination and for taking advantage of the patient monitoring and quality control that nurses provide [13]. It has also been noted that patients have greater satisfaction with family doctors [14].

The aim of this study is to describe the characteristics of patients that were diagnosed of the first episode of CAP among patients with or without diabetes in Spain.

## Methods

This is a retrospective observational study aimed at describing the epidemiological characteristics of patients over 18 years of age who suffered their first episode of CAP between January 2009 and December 2013 and were diabetic patients. As inclusion criteria, patients had to have been treated for at least one year by their PC doctor.

The diagnosis of CAP in Primary Care in Spain is based in the history and physical examination (the presence of cough, fever, tachycardia and cramps [15]) and must be supported by chest radiography and an elevation of reactive protein C [16].

The database used was the Database for Pharmacoepidemiological Research in Primary Care (Base de datos para la Investigación Farmacoepidemiológica en Atención Primaria, BIFAP) from the Spanish Agency for Medicine and Health Products (Agencia Española del Medicamento y Productos Sanitarios) [17], previously described in the literature [9]. Briefly, the BIFAP is one of the largest PC databases available; it aims to facilitate the development of pharmacoepidemiological studies to calculate the incidences and prevalences of diseases and investigate risk factors and prognoses using data from thousands of patients [18]. This database collects information from 5871 family doctors and paediatricians who are PC providers in the Spanish National Health System.

### Statistical analysis

Data were presented as median (IQR) or frequency (percentage) as appropriate. We carried out a descriptive analysis of the first episodes of CAP, in patients with or without diabetes as comorbidity. The relationship between different comorbidities or categorical variables and diabetes was tested using a Chi square test. Wilcoxon test was considered to test if there are differences between cohorts in terms of numeric variables. We performed all the analyses using R Software, Version 3.0.2. The significance level was set to 0.05.

Age was stratified into two categories, 18–59 and ≥ 60 years old based on that metabolism changes with age and is characteristically different after the age of 60, becoming generally slower, with a lower capacity for digestion and assimilation of macro- and micro-nutrients increasing the prevalence of diabetes after that age.

The information contained in the database was anonymized in order to prevent the identification of patient, physician and centre, in strict keeping with Spanish and European legislation on this matter.

## Results

A total of 51,185 patients diagnosed of the first episode of CAP were included in the study of which 8012 patients had diabetes. The median age was 76.1 years old (IQR: 66.0–83.0), and 59.3% were men. A 71.8% of the patients had two or more morbidity factors additionally to diabetes.

### Differences between those patients with or without diabetes

Demographic characteristics and comorbidities of diagnosis of CAP in patients with diabetes and in patients without diabetes are shown in Table 1 in Appendix. In patients who have diagnosis of CAP, there was a significant male predominance (59.3% for diabetes and 50.2% for no diabetes; *P*-value = 5.51 × 10^− 51^). Generally, patients with diabetes and CAP were significantly older than patients without diabetes (median [IQR]: 76.1 [66.0; 83.0] vs. 60.1 [42.1; 77.4]; *P*-value < 10^− 152^). An 86.1% of the diabetic patients had developed CAP while in non-diabetic patients a 50.2% have had an episode of CAP (*P*-value < 0.001) and had more coexisting chronic medical conditions and lifestyle risk factors. Specifically, diabetic patients with CAP had higher prevalence of heart disease (27.4% vs. 9.8%; *P*-value < 10^− 152^), kidney disease (9.4% vs. 3.1%; *P*-value = 1.07 × 10^− 152^),), COPD (18.6% vs. 10.0%; *P*-value = 1.63 × 10^− 109^), CVA (9.9% vs. 4.1%; *P*-value = 2.40 × 10^− 108^), anaemia (20.4% vs. 12.1%; *P*-value = 7.51 × 10^− 90^), arthrosis (13.3% vs. 7.3%; *P*-value = 1.1 × 10^− 13^), dementia (8.9% vs. 4.7%; *P*-value = 1.72 × 10^− 53^), liver disease (6.3% vs. 3.2%; *P*-value = 5.42 × 10^− 42^), depression (20.0% vs. 15.8%; *P*-value = 8.03 × 10^− 21^), dysphagia (1.6% vs. 1.2%; *P*-value = 0.001, Parkinson’s disease (2.7% vs. 1.5%; *P*-value = 1.1 × 10^− 13^). Tobacco exposure (*P*-value = 1.00 × 10^− 141^), dental problems (*P*-value = 4.95 × 10^− 13^), alcoholism (*P*-value = 5.55 × 10^− 8^), and social problems (*P*-value = 1.41 × 10^− 6^) were lifestyle factors more prevalent in those patients with diabetes and CAP.

Contrary, eating disorder (1.9% vs. 1.0%; *P*-value = 1.33 × 10^− 7^) and HIV (0.9% vs. 0.2%; *P*-value = 2.07 × 10^− 10^) were more prevalent in non-diabetic patients. There are no statistical significant differences between diabetic and no diabetic CAP cases regarding to medical conditions arthritis, asthma, multiple sclerosis and epilepsy.

In non-diabetic patients who develop CAP a 21.1% do not have any other risk factor associated and 31.3% had one more risk factor. In diabetic patients with CAP usually have 2 or more other risk factors associated.

### Age and sex analysis in diabetic patients

**Fig. 1:**
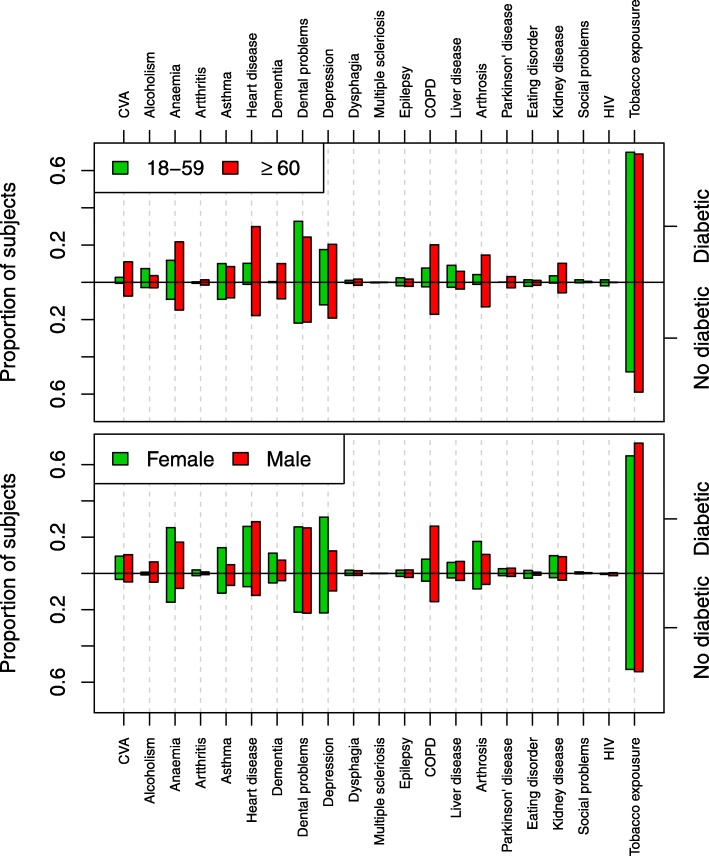
‘Differences between diabetic and no diabetic by sex and age.’ In this figure you can see the proportion of subjects who had every risk factor in each group: diabetic and no diabetic; by sex and age

A total of 86.1% of the diabetic patients with CAP were ≥ 60 years of age and some comorbidities were more prevalent in this age group (Figure 1): heart disease (29.9% vs. 10.2%; *P*-value = 3.53 × 10^− 39^), dementia (10.1% vs. 0.4%; *P*-value = 7.05 × 10^− 24^), COPD (20.2% vs. 7.6%; *P*-value = 1.35 × 10^− 21^), arthrosis (14.7% vs. 4.2%; *P*-value = 7.18 × 10^− 20^), CVA (11.0% vs. 2.7%; *P*-value = 2.90 × 10^− 16^), anaemia (21.7% vs. 11.8%; *P*-value = 4.13 × 10^− 13^), Parkinson’s disease (3.1% vs. 0.1%; *P*-value = 8.25 × 10^− 08^) and renal disease (10.2% vs. 3.5%; *P*-value = 7.65 × 10^− 12^) and arthritis (1.3% vs. 0.3%; *P*-value = 8.40 × 10^− 3^).

According to sex, we found that men with diabetes and CAP had a younger average age and a higher frequency of COPD (26.0% vs. 7.8%; *P*-value = 2.55 × 10^− 94^), alcoholism (6.3% vs. 0.7%; *P*-value = 1.56 × 10^− 35^), and smoking (71.8% vs. 64.8%; *P*-value = 3.18 × 10^− 11^). Diabetic men with CAP have no other risk factors associated (Fig. 1). No differences were found according to gender in the frequency of CVA, poor dental hygiene, dysphagia, multiple sclerosis, epilepsy, hepatitis, Parkinson disease, renal disease and HIV.

## Discussion

A significant number of diabetic patients have suffered from CAP. Diabetes is a risk factor for suffering CAP and there are other comorbidities and life-style factors associated to these conditions that can be controlled to diminish the risk of suffering CAP.

The average age of onset of the first episode of CAP is high, which may be due to improvements in diabetic control through PC clinics [19].

Patients are ≥60 years and diabetes have more prevalence of other comorbidities, which makes them more vulnerable to any infection such as CAP. Elderly people have weaker immunity system because of age, if they also have more comorbidities apart from diabetes, the control and immunization in this age group becomes more important. The higher incidence of diabetes in men coincides with the published literature [20], probably because in the latest population reports, women tend to have healthier lifestyles, and diabetes is related to characteristics such as obesity, smoking and a lack of dietary fruits and vegetables [21]. These differences in life-style factors between male and female are also present in this study as smoking and alcoholism. This could be the reason men have more risk to develop heart disease and COPD than women.

Importantly, almost 14% of patients that had DM and developed CAP were < 60 years of age. These patients could be of particular interest, as lifestyle interventions and vaccination could have an impact on reducing their risk of developing CAP.

Alcoholism, dental problems, social problems and tobacco exposure were lifestyle factors more prevalent in those patients with diabetes and CAP.

Patients with diabetes were older than without diabetes and majority male coinciding with the literature [22].

Those factors more presents in patients with diabetes than patients without diabetes are related with CAP such as: COPD, a known risk factor for CAP [23], depression is a mortality risk factor for patients admitted to the hospital with CAP in some groups [24], kidney disease is related to higher mortality in patients with diabetes and CAP [25], anaemia is an analytic finding in patients with pneumonia in the literature [26].

Lifestyle factors such as, tobacco expousure, social problems, dental problems and alcoholism have been associated with diabetes in patients with CAP. Smoking is the factor most often present in diabetic patients, and it is described as a risk factor for CAP [27] not only in smokers but also in passive smokers and ex-smokers [28], something to which the occurrence of a second episode of CAP can be attributed. Some experts propose pneumococcal vaccination for immunocompetent smokers [29]. Alcoholism is also describe as an increased risk in CAP and it’s more present in patients with diabetes [9, 30].Periodontal disease is a present factor and a risk factor for a worse trajectory of type II diabetes [31]. In addition, poor oral hygiene is related to the occurrence of CAP [32].

In non-diabetic patients who develop CAP almost one third had one more risk factor, but in diabetic patients with CAP usually have 2 or more other risk factors associated. They are complex patients with multimorbidity [33] who require personalized care [34] developed for them [35].

The coexistence of chronic pathologies in the same patient reduces their immune system’s strength, making them more susceptible to suffering a second case of CAP [36] because the risk of CAP increases with the presence of chronic diseases [37], producing a cumulative risk effect [38]. This reflects the clear importance of immunization in these patients [39] to prevent hospitalizations because patients with diabetes have a higher risk of hospital admission due to CAP [5, 40].

Finally, it is important to know the comorbidities and smoking and alcohol habits of the patients because bacterial aetiologies can differ based on these personal factors [41]. This influences treatment choices and therefore the patient’s prognosis and recovery from CAP.

The present study has potential limitations. We include diabetes type I and II and we did not consider patient treatment variables in order to indirectly stratify the degree of diabetic control that could have an influence in CAP [42, 43]. However, the BIFAP is one of the largest PC databases available [18], and the large sample of patients and variables available through it provides a good representation of the current population of diabetics who have had their first episodes of pneumonia in the context of a PC setting. This greatly strengthens the study.

## Conclusions

Patients with diabetes who developed an episode of CAP are male over 60 years old and have more risk factors associated than those patients without diabetes who suffer from CAP. Diabetes and those other risk factors associated could be reduced with an appropriate intervention, including vaccination to prevent the first and successive CAP episodes and the subsequent hospitalization in severe cases.

The burden of associated factors in patients with diabetes can produce an accumulation of risk. Health care professionals should know this for treating and control these patients in order to avoid complications.

## Data Availability

Most of the data generated or analysed during this study are included in this published article [and its additional file], although restrictions apply to the availability of some data, which were used under license for the current study, and so are not publicly available. Data are however available from the authors upon reasonable request and with permission of BIFAP database.

## Appendix

**Table 1:** Distribution of the variables in patients with and without diabetes

	Total (*n* = 51,185)	No diabetic (*n* = 43,173)	Diabetic (*n* = 8012)	P-value
Sex (%)				
Male	24,760 (48.4%)	21,502 (49.8%)	3258 (40.7%)	< 0.001
Female	26,425 (51.6%)	21,671 (50.2%)	4754 (59.3%)	
Age median (IQR)	63.8 (44.7; 79.0)	60.1 (42.1; 77.4)	76.1 (66.0; 83.0)	< 0.001
Age category (%)				
18–59	22,623 (44.2%)	21,513 (49.8%)	1110 (13.9%)	< 0.001
60≤	28,562 (55.8%)	21,660 (50.2%)	6902 (86.1%)	
CVA (%)	2545 (5.0%)	1751 (4.1%)	794 (9.9%)	< 0.001
Alcoholism (%)	1563 (3.1%)	1241 (2.9%)	322 (4.0%)	< 0.001
Anaemia (%)	6859 (13.4%)	5222 (12.1%)	1637 (20.4%)	< 0.001
Arthritis(%)	546 (1.1%)	451 (1.0%)	95 (1.2%)	0.285
Asthma (%)	4480 (8.8%)	3788 (8.8%)	692 (8.6%)	0.706
Heart disease (%)	6424 (12.6%)	4226 (9.8%)	2198 (27.4%)	< 0.001
Dementia (%)	2724 (5.3%)	2013 (4.7%)	711 (8.9%)	< 0.001
Dental problems (%)	11,381 (22.2%)	9352 (21.7%)	2029 (25.3%)	< 0.001
Depression (%)	8404 (16.4%)	6803 (15.8%)	1601 (20.0%)	< 0.001
Dysphagia (%)	625 (1.2%)	497 (1.2%)	128 (1.6%)	0.001
Multiple scleriosis (%)	91 (0.2%)	81 (0.2%)	10 (0.1%)	0.280
Epilepsy (%)	1009 (2.0%)	864 (2.0%)	145 (1.8%)	0.277
COPD (%)	5812 (11.4%)	4322 (10.0%)	1490 (18.6%)	< 0.001
Liver disease (%)	1891 (3.7%)	1384 (3.2%)	507 (6.3%)	< 0.001
Arthrosis (%)	4219 (8.2%)	3151 (7.3%)	1068 (13.3%)	< 0.001
Parkinson’s disease (%)	877 (1.7%)	660 (1.5%)	217 (2.7%)	< 0.001
Eating disorder (%)	897 (1.8%)	814 (1.9%)	83 (1.0%)	< 0.001
Kidney disease (%)	2075 (4.1%)	1323 (3.1%)	752 (9.4%)	< 0.001
Social problems (%)	157 (0.3%)	110 (0.3%)	47 (0.6%)	< 0.001
HIV	419 (0.8%)	401 (0.9%)	18 (0.2%)	< 0.001
Tobacco exposure (%)	28,700 (56.1%)	23,173 (53.7%)	5527 (69.0%)	< 0.001
Number of risk factors (%)				< 0.001
0	9628 (18.8%)	9106 (21.1%)	522 (6.5%)	
1	15,236 (29.8%)	13,498 (31.3%)	1738 (21.7%)	
2	12,336 (24.1%)	10,231 (23.7%)	2105 (26.3%)	
3	7675 (15.0%)	5927 (13.7%)	1748 (21.8%)	
> 3	6310 (12.3%)	4411 (10.2%)	1899 (23.7%)	

## Abbreviations

BIFAP: Database for Pharmacoepidemiological Research in Primary Care (Base de datos para la Investigación Farmacoepidemiológica en Atención Primaria)
CAP: Community Acquired Pneumonia
COPD: Chronic Obstructive Pulmonary Disease
CVA: Cerebrovascular Accident
HIV: Human Immunodeficiency Virus
PC: Primary Care
